# Risk factors for prolonged hypotension in patients with pheochromocytoma undergoing laparoscopic adrenalectomy: a single-center retrospective study

**DOI:** 10.1038/s41598-017-06267-z

**Published:** 2017-07-19

**Authors:** Shubin Wu, Weiyun Chen, Le Shen, Li Xu, Afang Zhu, Yuguang Huang

**Affiliations:** Department of Anesthesiology, Peking Union Medical College Hospital, Chinese Academy of Medical Sciences and Peking Union Medical College, Beijing, 100730 P.R. China

## Abstract

Prolonged hypotension during pheochromocytoma resection is a significant complication. We sought to investigate the predictors of prolonged hypotension in patients with pheochromocytoma undergoing laparoscopic adrenalectomy (LA). Patients with pheochromocytoma who underwent LA between 2012 and 2015 were surveyed. Patients were considered to have prolonged hypotension if they had a mean arterial blood pressure <60 mmHg or required ≥30 consecutive minutes of catecholamine support intraoperatively. Among 123 patients, 54 (43.9%) developed prolonged hypotension requiring ≥30 consecutive minutes of catecholamine support. Compared with patients with nonprolonged hypotension, those with prolonged hypotension had higher levels of urinary norepinephrine (*P* = 0.011), epinephrine (*P* < 0.001), and dopamine (*P* = 0.019) preoperatively, and a higher incidence of vital organ injury postoperatively (*P* = 0.039). Multivariate logistic analysis showed that independent predictors for prolonged hypotension were multiples of the normal reference upper limit value of urinary epinephrine (odds ratio, 1.180; 95% confidence interval, 1.035–1.345) and dopamine (odds ratio, 4.375; 95% confidence interval, 1.207–15.855). The levels of preoperative urinary epinephrine and dopamine are clinical predictors for prolonged hypotension in patients with pheochromocytoma undergoing LA. Using these parameters, clinicians can assess and manage this patient population more effectively.

## Introduction

Pheochromocytoma is a rare, catecholamine-secreting neuroendocrine tumor arising from chromaffin cells in the adrenal medulla. Although the clinical manifestation of pheochromocytoma varies, the predominant symptoms include episodic hypertension, headaches, sweating, and tachycardia^1^. With the developments in operative techniques, laparoscopic adrenalectomy (LA) has become the mainstay of therapy for patients with pheochromocytoma^2^.

Perioperative mortality has decreased dramatically in this population due to advances in preoperative treatment, surgical techniques, and anesthesia^1, 3^. Significant efforts have been made to prevent the occurrence of hypertensive episodes, especially during anesthesia induction, pneumoperitoneum creation, and adrenal gland manipulation^4, 5^. Nevertheless, hypotension episodes resulting from catecholamine withdrawal are difficult to avoid during pheochromocytoma resection and may lead to significant complications^6, 7^. These episodes generally necessitate continuous vasoconstrictors despite treatment with fluid replacement^4^. Another challenge during the postoperative management of patients includes vital organ injury, which is closely associated with sustained intraoperative hypotension during noncardiac surgery^8, 9^.

Various indices of hemodynamic instability have been associated with tumor size, as well as the plasma and urinary concentrations of catecholamines^2, 4, 10, 11^. However, to our knowledge, limited data exist on prolonged hypotension following tumor withdrawal as a single index. Hence, we sought to identify the clinical risk factors related to prolonged hypotension during LA in patients with pheochromocytoma. The relationship between intraoperative prolonged hypotension and postoperative vital organ injury was also evaluated in this population.

## Results

**Figure 1:**
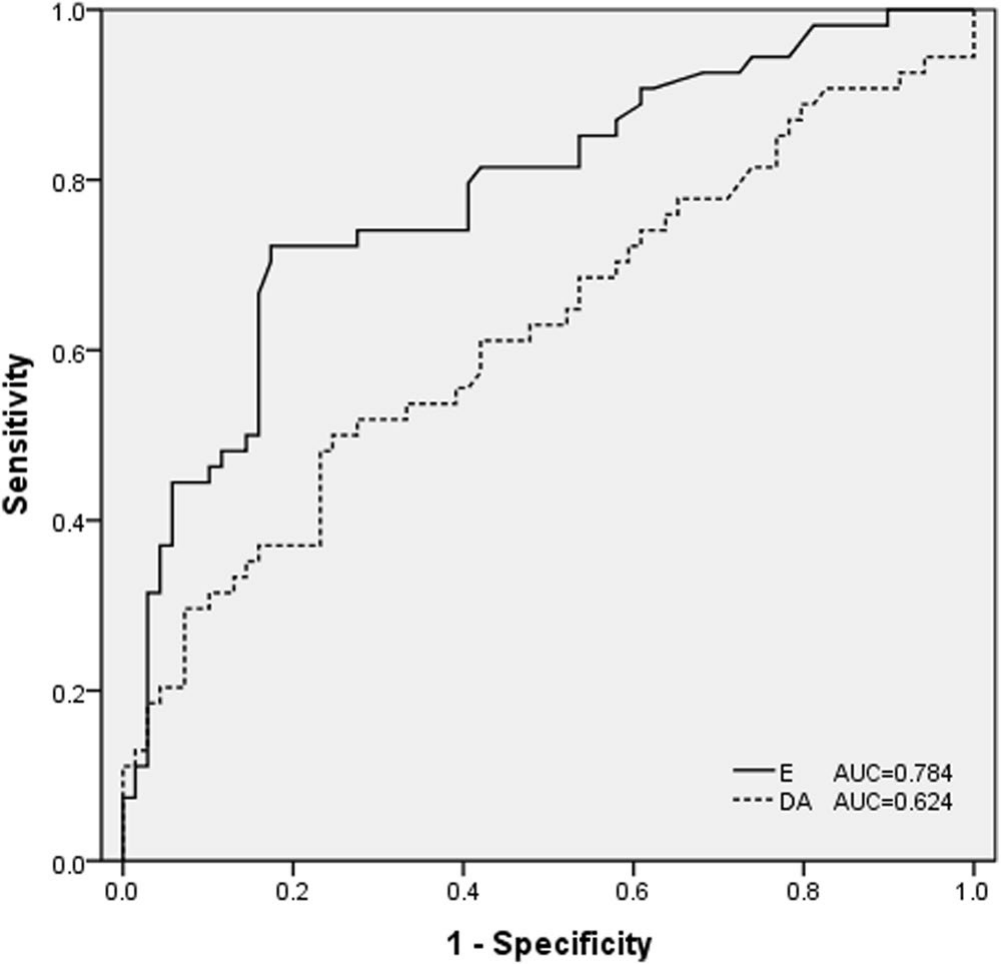
Receiver operating characteristic curve of multiples of the normal reference upper limit value of urinary E and DA. Cut-off points: multiples of the normal reference upper limit value of E, 0.5; multiples of the normal reference upper limit value of DA, 0.6. AUC, area under the curve; DA, dopamine; E, epinephrine.

Among 123 patients, 54 (43.9%) had intraoperative prolonged hypotension. These patients required continuous catecholamine support to maintain mean arterial blood pressure (MAP) ≥60 mmHg intraoperatively. The median duration of catecholamine infusion was 53 min (range, 32–217 min). The patients demographics and tumor characteristics are shown in Table 1. The fold changes of epinephrine, norepinephrine, and dopamine were greater in patients with prolonged hypotension than in those with nonprolonged hypotension. In addition, a greater proportion of patients with nonprolonged hypotension was asymptomatic, compared with those with prolonged hypotension. A lower proportion of patients with nonprolonged hypotension had diabetes than patients with prolonged hypotension. Table 2 shows that patients with prolonged hypotension required a longer duration of catecholamine support than those with nonprolonged hypotension. As shown in Table 3, patients with prolonged hypotension had a longer intubation time in the intensive care unit and length of hospitalization than those with nonprolonged hypotension. Compared with patients with nonprolonged hypotension, the incidence of postoperative vital organ injury was higher in patients with prolonged hypotension. In the multivariate analysis, multiples of the normal reference upper limit value of epinephrine and dopamine were independent predictors of prolonged hypotension during pheochromocytoma resection, with odds ratios of 1.180 (95% confidence interval, 1.035–1.345) and 4.375 (95% confidence interval, 1.207–15.855), respectively (Table 4). Receiver operating characteristic curve analysis was performed for each variable to identify the optimal cut-off point that correlated with prolonged hypotension during LA for patients with pheochromocytoma. The cut-off points for the multiples of the normal reference upper limit value of epinephrine and dopamine were 0.5 and 0.6, respectively (Fig. 1).

**Table 1:** Patient demographics and tumor characteristics.

	Nonprolonged hypotension (n = 69)	Prolonged hypotension (n = 54)	*P* value
Age (years)	45 ± 14	47 ± 13	0.594
Sex, men (%)	27 (39)	21 (39)	0.978
Body mass index (kg/m^2^)	24 ± 3	23 ± 3	0.324
Asymptomatic, n (%)	16 (23)	5 (9)	0.042
ASA	2 (1–3)	2 (2–3)	0.096
Comorbidity, n (%)			
Cardiovascular disease	2 (3)	4 (7)	0.465
Hypertension	26 (38)	18 (33)	0.618
Diabetes mellitus	9 (13)	17 (32)	0.013
Tumor size (mm)	44 ± 16	49 ± 19	0.086
Tumor location, n (%)			
Bilateral	3 (4)	6 (11)	0.280
Multiple of the normal reference upper limit value			
E	0.4 (0.1–18.5)	0.6 (0.3–53.8)	<0.001
NE	1.2 (0.2–30.3)	3.1 (0.4–26.1)	0.011
DA	0.5 (0.2–1.4)	0.6 (0–4.2)	0.019
Phenoxybenzamine (mg/day)	20 (0–80)	28 (10–65)	0.326
Duration of *α* blockade (day)	32 (0–130)	33 (8–122)	0.474
Preoperative medications, n (%)			
*α* blockade	63 (91)	54 (100)	0.072
Selective *α* blockade	6 (9)	6 (11)	0.654
*β* blockade	13 (19)	15 (28)	0.241
Calcium channel blockade	3 (4)	3 (6)	1.000

ASA, American Society of Anesthesiologists Physical Status Classification System. NE, norepinephrine; E, epinephrine; DA, dopamine.

**Table 2:** Intraoperative variables.

	Nonprolonged hypotension (n = 69)	Prolonged hypotension (n = 54)	*P* value
Minimum MAP (mmHg)	71 (60–88)	67 (46–85)	0.027
Catecholamine support (min)	0 (0–28)	53 (32–217)	<0.001
Total fluid intake (mL)	3388 ± 1225	3651 ± 1248	0.242
Vasodilators, n (%)	51 (73.9)	39 (72.2)	0.834
Urinary output (mL)	300 (0–1600)	500 (0–2000)	0.239
Pneumoperitoneum (mmHg)	14.2 ± 0.5	14.3 ± 0.5	0.376
Surgical approach, n (%)			0.910
Transperitoneal	4 (6)	2 (4)	
Retroperitoneal	65 (94)	52 (96)	
Blood loss (mL)	100 (10–1600)	100 (10–2500)	0.797
Anesthetic time (min)	160 (85–300)	165 (95–333)	0.521
Operative time (min)	115 (40–260)	113 (15–240)	0.826

MAP, mean arterial blood pressure.

**Table 3:** Postoperative parameters.

	Nonprolonged hypotension (n = 69)	Prolonged hypotension (n = 54)	*P* value
Intubation time (hour)	12 (0–28)	18 (0–48)	0.012
ICU stay (hour)	24 (13–73)	15 (15–99)	0.210
Length of hospitalization (day)	14 (6–49)	17 (7–86)	0.033
Vital organ injury^a^, n (%)	2 (2.9)	8 (14.8)	0.039

ICU, intensive care unit.

^a^Vital organ injury was defined as acute kidney injury and cardiac injury.

**Table 4:** Uni- and multivariate analyses for predictors of prolonged hypotension.

Variables	Univariate analysis		Multivariate analysis	
	*P* value	OR (95% CI)	*P* value	OR (95% CI)
Asymptomatic	0.048	2.958 (1.008–8.683)	0.769	1.197 (0.360–3.985)
Diabetes mellitus	0.015	3.063 (1.238–7.578)	0.381	1.640 (0.542–4.964)
ASA	0.079	1.861 (0.931–3.722)	0.207	1.679 (0.750–3.757)
NE^b^	0.028	1.086 (1.009–1.169)	0.116	1.066 (0.984–1.155)
E^b^	0.004	1.207 (1.062–1.371)	0.013	1.180 (1.035–1.345)
DA^b^	0.017	4.159 (1.295–13.360)	0.025	4.375 (1.207–15.855)
Tumor size	0.088	1.018 (0.997–1.040)	0.578	0.993 (0.967–1.019)

ASA, American Society of Anesthesiologists Physical Status Classification System; NE, norepinephrine; E, epinephrine; DA, dopamine; OR, odds ratio; CI, confidence interval.

^b^Multiple of the normal reference upper limit value.

## Discussion

In this study, we found that prolonged hypotension occurred in 54 (43.9%) patients and required continuous catecholamine support to maintain MAP ≥60 mmHg intraoperatively. Our multivariate analysis demonstrated that multiples of the normal reference upper limit value of epinephrine and dopamine were independent predictors of prolonged hypotension during LA.

Intraoperative hypotension remains a common complication despite adequate preoperative preparation^6, 7, 12^. Previous reports have shown that intraoperative hypotension occurs in 39–48% of patients with pheochromocytoma^6, 7^. In this study, 43.9% of patients needed catecholamine support after pheochromocytoma resection to maintain MAP ≥60 mmHg. This is consistent with the results of previous studies.

Managing hypotension can represent a substantial challenge in a subset of cases. Management of hypotension begins with intravascular volume expansion. Continuous vasoconstrictors are also required in patients with severe hypotension during pheochromocytoma resection^6, 7^. Generally, chronic hypovolemia and abrupt catecholamine withdrawal are thought to be the main causes of hypotension after pheochromocytoma resection^2, 4^. Furthermore, hypotension can be ascribed to down-regulation of *α*- and *β*-adrenoceptors resulting from long-term plasma catecholamine elevation^13, 14^.

In the present study, we found that preoperative urinary epinephrine was associated with prolonged hypotension during LA in patients with pheochromocytoma. Catecholamines exceeding in majority of patients with pheochromocytoma exert their effects by adrenoceptors^15^. Epinephrine and norepinephrine have overlapping but distinct effects on *α*- and *β*-adrenoceptors in the human body. In low doses, epinephrine acts predominantly on the peripheral *β*_1_- and *β*_2_-adrenoceptors. However, with increasing doses of epinephrine, the *α*_1_-adrenoceptor–mediated vasoconstrictor effect predominates. Norepinephrine acts predominantly on *α*-adrenoceptors to induce peripheral vasoconstriction, and has almost no effect on *β*-adrenoceptors.

Although hypertension is the most common symptom in patients with pheochromocytoma, hypotension is frequently seen in patients with epinephrine-secreting tumors even before surgery^16, 17^. Furthermore, excessive circulating epinephrine likely decreases cardiac contractility by down-regulating *β*-adrenoceptors in the heart^16, 18^. In a previous study, investigators found that acute left cardiac dysfunction due to chronic elevated concentrations of epinephrine was the root cause of hypotension and circulatory collapse after pheochromocytoma resection^19^. Thus, the depression of cardiac contraction force may be induced, and aggressive volume expansion may increase the workload on the left heart^16, 18, 19^.

According to the underlying actions of epinephrine and norepinephrine, catecholamine infusion improves low cardiac output, especially in patients with epinephrine-secreting pheochromocytoma, although both epinephrine and norepinephrine have been suggested to be related to postresection hypotension^15, 16, 18, 20^. Hypotension after pheochromocytoma resection has been thought to be partially attributed to hypovolemia. However, a prospective study suggested that preoperative fluid therapy had no impact on postresection hypotension^21^, indicating that intraoperative hypotension may be largely due to down-regulation of *β*-adrenoceptors. We consider that the preoperative urinary levels of epinephrine are a robust risk factor for prolonged hypotension in patients with pheochromocytoma undergoing LA.

This study also suggests that preoperative urinary dopamine is one of the predictors of prolonged hypotension during laparoscopic tumor resection. Dopamine-secreting tumors are rare compared to norepinephrine-secreting tumors, especially for dopamine-predominant pheochromocytoma. Patients with dopamine-secreting pheochromocytoma tend to be nonspecific and normotensive preoperatively^22^. This phenomenon can be explained by several mechanisms. In particular, dopamine offsets the vasoconstrictor effect of norepinephrine through D1 receptors in a dose-dependent manner. Dopamine suppresses norepinephrine release from neurons by acting on the presynaptic D2 receptors^23^. The same mechanisms may worsen the hypotensive scenario arising from catecholamine withdrawal during LA in patients with pheochromocytoma.

It should also be noted that dopamine lacks the *α*-adrenoceptor affinity for norepinephrine and epinephrine^23^. In dopamine-producing tumors, there is little *α*-adrenoceptor stimulation excess. Researchers have speculated that the *α* blockade may lead to hypotension or even cardiovascular collapse^24^. Therefore, preoperative antihypertensive drugs are not recommended in patients with exclusively dopamine-secreting paraganglioma or pheochromocytoma. In this study, 14 patients had dopamine-producing pheochromocytomas, including three patients with exclusively dopamine-secreting tumors. Preoperative preparation was empirically used in all of these patients, and 11 of them, including two cases secreting only dopamine, developed prolonged hypotension. However, this result was not necessarily attributed to the preoperative *α*-blockade preparation, because dopamine itself was an independent predictor of prolonged hypotension. Furthermore, *α* and *β* blockades were empirically prescribed before surgery in one previously reported case with no adverse effects^25^. In another case where preoperative *α* blockade was not administered, the patient had drastic intraoperative hypertension and hypotensive episodes^26^. Thus, the relationship between preoperative antihypertension medication and intraoperative hypotension may need to be further evaluated in patients with dopamine-secreting tumors.

Intraoperative hypotension is common during surgery and may be a significant determinant of postoperative vital organ injury—mainly acute kidney injury and cardiac injury—and other complications^8, 9, 27^. In this study, patients with prolonged hypotension experienced longer intubation time and length of hospitalization and had a higher incidence of postoperative vital organ injury than patients without prolonged hypotension. Our findings validate the previously published research by Sun *et al*.^8^ and Walsh *et al*.^9^. Because blood pressure is one of very few adjustable intraoperative risk factors for postoperative adverse outcomes, it is important to prevent and correct hypotension in a timely manner during adrenalectomy in patients with pheochromocytoma, especially in those with epinephrine- and dopamine-secreting tumors.

It is noteworthy that most of the patients in this study received phenoxybenzamine for preoperative medical preparation. Non-selective *α* blockade has been shown to relate with hypotensive episodes after tumor removal owing to the covalent binding of the drug to the *α* adrenoceptors. This situation may not improve until the *α* adrenoceptors are regenerated on the cell surface, which may not occur until several hours after tumor resection^28, 29^. As a result, non-selective *α* blockade can lead to persistent intraoperative hypotension requiring fluid therapy and continuous catecholamine infusion, although it works better to control hypertensive crises^30, 31^. So far, several studies have confirmed the relationship between phenoxybenzamine and perioperative hypotension in patients with pheochromocytoma^30–32^. Consequently, the effect of phenoxybenzamine on intraoperative hypotension in this study can not be ruled out, despite the fact that the ratio of phenoxybenzamine usage did not statistically significantly differ between the two groups.

This study has several strengths. We surveyed patients who underwent LA very recently (2012–2015), which allowed for the homogeneous distribution of individual patients’ physiology in response to the same anesthetic and surgical techniques. Furthermore, the invasive blood pressure was monitored electronically and recorded during pheochromocytoma resection in our center, which should decrease the potential bias associated with estimating these values. In addition, the relatively large sample size rendered the multivariable modelling stable, thus enabling the detection of associations between preoperative urinary catecholamines and intraoperative prolonged hypotension.

However, we also acknowledge that our study has several limitations. First, the retrospective design of our study may affect the quality of the data. Second, intraoperative hemodynamic variables were collected from electronic recording updated every 5 min, which may have limited the accuracy of our counting parameters. Third, the urinary and plasma levels of metanephrine and normetanephrine were not routinely measured in our center. Future research is needed to evaluate the relationship between these two parameters and intraoperative hypotension. Finally, the results of the multivariate analysis pertaining to dopamine should be interpreted with caution, owing to the wide 95% confidence interval originated from the rarity of dopamine-secreting tumors. The relationship between preoperative medication and intraoperative hypotension also needs to be further elucidated, especially in patients with dopamine-secreting pheochromocytoma.

## Conclusion

In conclusion, intraoperative prolonged hypotension is a relatively frequent complication (43.9%) in patients undergoing pheochromocytoma resection. Intraoperative prolonged hypotension is likely to be related to postoperative vital organ injury in pheochromocytoma patients. Furthermore, multiples of the normal reference upper limit value of urinary epinephrine (>0.5) and dopamine (>0.6) are independent predictors of prolonged hypotension in patients with pheochromocytoma undergoing LA.

## Methods

### Study subjects

Ethical approval (Ethical Committee No. S-K157) was provided by the Institutional Research and Ethics Committee of the Peking Union Medical College Hospital in Beijing, China (Chairperson Prof Long-Cheng Li) on 21 September 2016; the need for informed consent from all eligible patients was waived.

We retrospectively surveyed 127 patients with pheochromocytoma who underwent LA at our center between December 26, 2012, and October 9, 2015. Retroperitoneal laparoscopic surgery was preferred and performed for most patients. Four patients were excluded from the study because of missing data. All patients enrolled were identified as having pheochromocytoma from the postoperative pathology results.

In our institution, all patients diagnosed with pheochromocytoma via biochemical tests and imaging examinations were treated with phenoxybenzamine or doxazosin at least 2 weeks preoperatively. Metoprolol was added to control episodes of tachycardia if necessary. During the period of preoperative medication, oral hydration was encouraged, and patients were admitted for intravenous fluids 2 days before tumor resection. The criteria for efficacy include blood pressure <165/80 mmHg 2 days before surgery and standing blood pressure >80/45 mmHg. All patients received general anesthesia for the surgery. Intraoperatively, the invasive blood pressure and heart rate were automatically recorded. Intravenous vasodilators (sodium nitroprusside, phentolamine, and nitroglycerin) and esmolol were administered to control undesirable increases in blood pressure and heart rate, respectively. All patients were transferred to the intensive care unit postoperatively. Prolonged hypotension during LA was managed using fluid replacement therapy and continuous catecholamine administration.

### Laboratory and clinical parameters and term definitions

Data on the patient demographics, clinical history, laboratory analysis, and intraoperative details were collected. Patients were considered to have prolonged hypotension if they had a MAP <60 mmHg or required ≥30 consecutive minutes of catecholamine support intraoperatively^33^. Vital organ injury was defined as acute kidney injury and cardiac injury. According to the Acute Kidney Injury Network threshold, patients were diagnosed with acute kidney injury if there was a 50% relative or 0.3 mg/dL absolute increase in creatinine from the preoperative value during the first two postoperative days^34^. Myocardial injury was defined as a postoperative cardiac enzyme concentration within 7 days of surgery that was greater than or equal to the suggested necrosis limit for troponin T and greater than the upper limit of normal for creatinine kinase MB^35^.

### Statistical analysis

The Mean (±standard deviation) and median (range) values were used to express normally and non-normally distributed continuous data, respectively. Comparisons between groups were conducted with the independent sample *t*-test for normally distributed variables, Mann-Whitney U test for non-normally distributed variables, and Chi square test for categorical variables. Multivariate logistic regression was used to determine factors associated with prolonged hypotension during LA in patients with pheochromocytoma. Using receiver characteristic operating curve, we calculated the cut-off points for the clinical risk factors associated with prolonged hypotension. Parameters with a *P* value < 0.10 in the univariate analysis were entered into a multivariate logistic regression analysis. Statistical significance was defined as *P* < 0.05. All statistical analyses were performed with SPSS version 19.0 (IBM, SPSS, Inc.).
